# Developing and testing a produce prescription implementation blueprint to improve food security in a clinical setting: a pilot study protocol

**DOI:** 10.1186/s40814-024-01467-7

**Published:** 2024-03-23

**Authors:** Hannah E. Frank, Linda E. Guzman, Shivani Ayalasomayajula, Ariana Albanese, Brady Dunklee, Matthew Harvey, Kelly Bouchard, Maya Vadiveloo, Amy L. Yaroch, Kelli Scott, Alison Tovar

**Affiliations:** 1 Department of Psychiatry and Human Behavior, The Warren Alpert Medical School of Brown University, Providence, RI USA; 2 Department of Behavioral and Social Sciences, Brown University School of Public Health, Providence, RI USA; 3 Integra Community Care Network, Providence, RI USA; 4 Care New England Health System, Providence, RI USA; 5 Department of Nutrition and Food Science, University of Rhode Island, Kingston, RI USA; 6 Gretchen Swanson Center for Nutrition, Omaha, NE USA; 7 Department of Medical Social Sciences, Northwestern University Feinberg School of Medicine, Chicago, IL USA

**Keywords:** Food insecurity, Produce prescriptions, Implementation science, Implementation blueprint, Protocol

## Abstract

**Background:**

Food insecurity is common in the United States, especially in Rhode Island, where it affects up to 33% of residents. Food insecurity is associated with adverse health outcomes and disproportionally affects people from minoritized backgrounds. Produce prescription programs, in which healthcare providers write “prescriptions” for free or reduced cost vegetables, have been used to address food insecurity and diet-related chronic disease. Although there is growing evidence for the effectiveness of produce prescription programs in improving food security and diet quality, there have been few efforts to use implementation science methods to improve the adoption of these programs.

**Methods:**

This two-phase pilot study will examine determinants and preliminary implementation and effectiveness outcomes for an existing produce prescription program. The existing program is funded by an Accountable Care Organization in Rhode Island and delivered in primary care practices. For the first phase, we conducted a formative evaluation, guided by the Consolidated Framework for Implementation Research 2.0, to assess barriers, facilitators, and existing implementation strategies for the produce prescription program. Responses from the formative evaluation were analyzed using a rapid qualitative analytic approach to yield a summary of existing barriers and facilitators. In the second phase, we presented our formative evaluation findings to a community advisory board consisting of primary care staff, Accountable Care Organization staff, and staff who source and deliver the vegetables. The community advisory board used this information to identify and refine a set of implementation strategies to support the adoption of the program via an implementation blueprint. Guided by the implementation blueprint, we will conduct a single-arm pilot study to assess implementation antecedents (i.e., feasibility, acceptability, appropriateness, implementation climate, implementation readiness), implementation outcomes (i.e., adoption), and preliminary program effectiveness (i.e., food and nutrition security). The first phase is complete, and the second phase is ongoing.

**Discussion:**

This study will advance the existing literature on produce prescription programs by formally assessing implementation determinants and developing a tailored set of implementation strategies to address identified barriers. Results from this study will inform a future fully powered hybrid type 3 study that will use the tailored implementation strategies and assess implementation and effectiveness outcomes for a produce prescription program.

**Trial registration:**

Clinical trials: NCT05941403, Registered June 9, 2023.

**Supplementary Information:**

The online version contains supplementary material available at 10.1186/s40814-024-01467-7.

## Background

The COVID-19 pandemic ended years of declining rates of food insecurity in the United States [1]. Food insecurity, which refers to unreliable access to sufficient and adequate food to meet one’s needs [2], is at an all-time high with 10.2% of the population living with food insecurity nationally [3]. Food insecurity is associated with suboptimal diets [4], increased risk for cardiovascular morbidity and mortality, and behavioral health problems [5, 6]. People with minoritized racial/ethnic identities and people with lower levels of socioeconomic status have been particularly impacted by food insecurity, as well as greater COVID-19 symptom severity and hospitalization [7]. Additionally, individuals with diet-related diseases associated with food insecurity (e.g., diabetes, obesity) were more likely to experience severe outcomes of COVID-19, including increased hospitalization [8]. It is worth noting that the term nutrition security has been brought to the forefront in recent years to highlight the importance of food quality in addition to food access. Nutrition security considers the nutritional value, affordability, accessibility, and safety of foods that promote well-being, with a focus on equity. While the pandemic exacerbated food and nutrition security, it also led to greater recognition of how local food systems could be leveraged in creative ways to get food to households [9, 10], especially among minoritized communities. Alleviating food and nutrition insecurity will require tackling the correlates of poverty while designing sustainable interventions that increase access and availability to nutrient-rich foods (e.g., vegetables) that are culturally informed.

There is growing evidence and increased attention with the recent White House Conference on Hunger, Nutrition, and Health on the importance of using “food as medicine” approaches to address food and nutrition insecurity, prevent associated negative health outcomes, and reduce healthcare costs [11, 12]. One such program involves the use of produce prescriptions that enable healthcare providers to identify patients at risk for food insecurity and/or cardio-metabolic disease and write prescriptions for vegetables [6]. This model has quickly grown, partly due to the Gus Schumacher Nutrition Incentive Program (GusNIP), which was authorized in the 2018 Farm Bill and formally funded produce prescription programs for the first time [13]. The predecessor to GusNIP, the Food Insecurity Nutrition Incentive (FINI) Program, funded nutrition incentive (but not produce prescription) programs. There is increasing evidence that produce prescriptions reduce food and nutrition insecurity and promote diet quality and health outcomes in minoritized populations [14–16]. A 2023 study of 22 produce prescription programs in the United States found that participation in such programs was associated with improvements in fruit and vegetable intake, food insecurity, and self-reported health status among both adults and children [17]. Adult participants with poor cardiometabolic health also experienced improvements in glycated hemoglobin, blood pressure, and body mass index [18].

Despite the growing evidence, there is variability in the effectiveness of produce prescription programs, and many barriers remain to successful scale-up across settings [19]. For example, many programs require participants to pick up the produce at a specific location, leading to transportation challenges [20]. Given that many of the beneficiaries of these programs are from low-income and culturally diverse backgrounds, it is possible that participants vary in their knowledge and use of certain vegetables. Such realities lead to decreased reach and participation of patient populations, especially those from underserved communities that stand to benefit from these programs. Healthcare staff also report having limited time to receive training, deliver screenings, and make patient referrals to produce prescription programs, resulting in variable implementation in healthcare settings [21–23]. Some programs are paired with nutrition education to improve health outcomes; however, these educational components fail to overcome these key transportation and time barriers. A recent report highlighted key research recommendations to support the expansion of produce prescriptions nationally, including the need for studies that involve key community members and assess both implementation and program effectiveness outcomes [24].

### A produce prescription program in Rhode Island

In the state of Rhode Island, food insecurity is prevalent (up to 33% of the population) [25]. Integra Community Care Network (Integra) is the Accountable Care Organization (ACO) of Care New England Health, Rhode Island Primary Care Physicians Corporation, and South County Health. Integra represents a network of more than 100 primary care practices. In 2019, Integra submitted an initial proposal for funding to support a produce prescription (VeggieRx) program in Rhode Island in response to the high rates of food insecurity; the program first launched in 2020. The program requires practice staff and providers to identify families with food insecurity and/or diet-related illnesses during primary care appointments using clinical judgment or with a 12-item screener that assesses health-related social needs including food insecurity. Identified patients complete an intake with practice staff to determine eligibility. Eligible patients are then referred to Southside Community Land Trust (SCLT), a local nonprofit that aggregates and packs produce from gardeners and small farmers who grow vegetables in environmentally sustainable ways on SCLT-managed land. SCLT also manages the format of delivery, including pickup or delivery. The VeggieRx program takes place biweekly from July to November of each year. Each bagged share contained 5–6 varieties of vegetables and herbs, weighing approximately 3–6 pounds, with an enrolled member of a household receiving one bagged share per delivery. Table 1 shows the number of participants, households, and practices involved in the program each year since the program’s initiation in 2020. Despite the program’s success in reaching a yearly average of approximately 34 households experiencing food insecurity, the program aims to continue to grow, with a goal of reaching approximately 50 households in 2023.

**Table 1:** VeggieRx enrollment by year

	**2020**	**2021**	**2022**
**Pickup/delivery**	Pickup only	Home delivery only	Home delivery only
**Total vegetable shares delivered (** ***N*** **)**	587	602	705
**Individuals receiving vegetables (** ***N*** **)**	119	147	144
**Households receiving vegetables (** ***N*** **)**	28	38	37
**Practices referring patients (** ***N*** **)**	1	3	4

Since its initiation, the program has been perceived positively by patients but also has faced challenges in achieving broader adoption including consistent screening and referrals by providers and clinical staff to ensure that eligible patients are offered the program. Implementation science focuses on identifying and addressing barriers to delivering an intervention in a clinical setting through research-supported implementation strategies [26]. Thus, consistent with an implementation science approach, a crucial next step in scaling up the VeggieRx program is to formally evaluate the barriers and facilitators that impact successful adoption and the optimal implementation strategies needed to enhance scale-up and retention. We do this by referencing the Consolidated Framework for Implementation Research 2.0 (CFIR 2.0 [27]). As a determinant framework [28], the CFIR 2.0 helps elucidate factors that influence implementation outcomes. The CFIR 2.0 assesses characteristics of the intervention, the inner setting in which the intervention is implemented (i.e., the primary care practices), and the outer setting in which the intervention resides (e.g., state policies). The development and preliminary testing of implementation strategies are needed to bolster the VeggieRx program’s adoption and impact in real-world settings, which is expected to ultimately impact participant health outcomes.

### Study aims

The *specific aims* of this pilot study [29] are as follows:

1. *Phase 1*: Engage in a formative evaluation to identify, using an implementation science determinant framework (the CFIR 2.0), barriers, facilitators, and current implementation strategies for the existing VeggieRx program with participating patients and providers/staff.
2. *Phase 2*: In response to identified barriers and facilitators, and together with community advisory board partners, develop a set of implementation strategies (i.e., an implementation blueprint) guided by the Expert Recommendations for Implementing Change Project (ERIC [30]) to support uptake of the VeggieRx program.
3. *Phase 2*: Conduct a pilot VeggieRx implementation study to assess both implementation outcomes (primary outcome: adoption; secondary outcomes: feasibility, acceptability, appropriateness, implementation climate, implementation readiness) and preliminary program effectiveness outcomes (primary outcome: food security; secondary outcome: depression symptoms).

This two-phase study will consider perspectives from VeggieRx providers/staff and patient recipients to modify implementation practices. The first phase includes Aim 1 and focused on formative evaluation of existing VeggieRx practices. The second phase includes Aims 2 and 3 and involves the development and piloting of an implementation blueprint to improve implementation of the VeggieRx program.

## Methods

### Study design

This two-phase study will: (1) develop an implementation blueprint of community advisory board (CAB)-informed strategies designed to address current barriers to implementating a produce prescription program (VeggieRx) and (2) conduct a pilot trial of these implementation strategies. Pilot outcomes will include adoption, feasibility, acceptability, and appropriateness of the VeggieRx program, as well as implementation climate and implementation readiness. We will also assess preliminary program effectiveness. These findings will inform a future hybrid type 3 effectiveness-implementation study. We use the Lancaster and Thabane guidelines [31] for reporting non-randomized pilot and feasibility studies, which represent an adapted version of the Consolidated Standards of Reporting Trials (CONSORT) [32, 33], as well as the Standards for Reporting Implementation Studies (STaRI) [34]. All study procedures and materials have been approved by Brown University’s Institutional Review Board.

### Setting

Our research team established a partnership with Integra, who oversees the VeggieRx program, and with SCLT, who provides fresh produce and manages the VeggieRx program food delivery. Currently, four Integra primary care practices are involved in the VeggieRx program; these include internal medicine, family medicine, and pediatric practices. The participating practices reach households from varying socioeconomic, racial, and ethnic backgrounds. In Phase 1, the four practices that most recently participated in the VeggieRx program were invited to participate. New practices that elected to participate in the 2023 VeggieRx program were also invited to participate in Phase 2 of the study. All practices were informed of plans for data collection, including contacting patients and individual clinicians to complete qualitative interviews and quantitative surveys (Phase 1), as well as newly developed VeggieRx implementation strategies (Phase 2). Participation in the VeggieRx program for practices and patients was not contingent on study participation.

### Phase 1 (Aim 1: formative evaluation to identify barriers, facilitators, and current VeggieRx implementation strategies)

#### Phase 1: formative evaluation participants

Healthcare providers and staff at practices participating in the VeggieRx program (*N* = 5) and patients who participated in the VeggieRx program (*N*= 9) were selected as the target populations for this study. Our sampling of five or more participants per informant category is in line with recommendations for achieving thematic saturation [35]. Healthcare providers and staff included anyone employed by each practice and involved in patient care, including physicians, nurses, medical assistants, and other members of the clinical team. Participants were eligible to participate if they were: (1) 18 years of age or older, (2) a healthcare provider or staff member in a practice participating in VeggieRx (regardless of whether they actually referred patients) *or* a recipient of VeggieRx, and (3) fluent in English and/or Spanish. Inclusion criteria were minimal to maximize the number and variability of participants. Patients who participate in the VeggieRx program were identified by their providers as eligible for the program as described above.

##### Community Advisory Board (CAB)

A group of community partners formed a CAB to provide input throughout the study. The CAB was initially convened during Phase 1 and played a central role in developing the implementation blueprint (Phase 2 Aim 2). CAB members were eligible but not required to provide data through completion of interviews in Phase 1 and/or surveys in Phase 2. The CAB (*N* = 6 members) consisted of Integra ACO staff, staff from SCLT, and staff from participating practices. Three study research team members also attended CAB meetings. Four of the CAB members were also research participants and completed interviews in Phase 1. Aligned with best practices [36], CAB members met with the research team to review roles, expectations, and communication plans for CAB participation. CAB meetings occurred three times over the course of the study, as described in more detail in the procedures section below.

#### Phase 1: recruitment

Providers and administrative staff members were primarily recruited through Integra. An Integra representative briefed on the study’s eligibility criteria contacted individuals affiliated with the care network to inform them about the study and collected contact information from those who expressed interest in participating and who verbally agreed to be contacted.

Patients enrolled in the VeggieRx program were recruited with the aid of SCLT staff who communicated with patients about their vegetable shares. SCLT staff shared study information with patients via text messages, including a link where they could sign up to participate. Recruitment flyers were printed and packaged with produce deliveries. The text messages and flyers were available in English and Spanish and distributed according to patients’ known language preference.

#### Phase 1: measures

A summary of the schedule of study measures and time points is provided in Table 2.

**Table 2:** Study measures

**Measure**	**CFIR 2.0 Outcomes Addendum Construct**	**Study period**		
		***Phase 1***	***Phase 2: baseline assessment***	***Phase 2:*** ***6-month follow-up assessment***
***Demographics*** ***(Pt, Pr)***	N/A	X^a^	X	
	**Implementation determinants and outcomes**			
***Acceptability of intervention*** ***measure (Pr)***	Acceptability		X	X
***Evidence-Based Practice Attitudes Scale (Pr)***	Acceptability (attitudes)	X		X
***Patient engagement (Pt)***	Acceptability		X (biweekly)	
***Number of patients enrolled in VeggieRx (St)***	Adoption		X	X
***Intervention appropriateness measure (Pr)***	Appropriateness		X	X
***Qualitative interviews (Pt/Pr)***	Determinants	X		
***Open-ended written response question***	Feasibility and acceptability			X (Pr only)
***Feasibility of Intervention Measure (Pr)***	Feasibility		X	X
***Participant enrollment (St)***	Feasibility		X	X
***Implementation Climate Scale (Pr)***	Implementation climate	X		X
***Organizational Readiness for Implementing Change (Pr)***	Implementation readiness		X	X
	**Innovation (effectiveness) outcomes**			
***USDA measure (Pt)***	Effectiveness: Food security		X	X
***Nutrition security measure (Pt)***	Effectiveness: Nutrition security		X	X
***Dietary Screener Guide (Pt)***	Effectiveness: Fruit and vegetable intake		X	X
***Patient Health Questionnaire-2 (Pt)***	Effectiveness: Depression		X	X

All measures will be available in Spanish and English

*Abbreviations*: *Pt* patient, *Pr* provider/staff, *St* study team

^a^Demographics questionnaires in Phase 1 included two items about food insecurity and four items about nutrition security

##### Qualitative interviews

Semi-structured interview guides were used to collect formative evaluation data. Interviews took place at the end of the 2022 VeggieRx delivery season (i.e., October-December 2022) and lasted 20–30 min. The interview guides consisted of questions that assessed barriers and facilitators to implementation of VeggieRx across the five domains of the CFIR 2.0, and an implementation science framework focused on determinants of new practice implementation, including the following: (1) the VeggieRx intervention; (2) the individuals involved in VeggieRx implementation, including VeggieRx recipients; (3) the inner setting of the practices where VeggieRx is implemented; (4) the outer setting in which VeggieRx is implemented; and (5) the VeggieRx implementation process. Semi-structured interviews also included questions about current VeggieRx implementation strategies and provider/patient perceptions of each strategy’s effectiveness. See Additional file 1 for the patient and staff interview guides.

##### Quantitative measures

Qualitative interview data were supplemented by quantitative surveys. Providers and staff completed reliable measures that assessed *provider attitudes* and *implementation climate*, including the Evidence-Based Practice Attitudes Scale (EBPAS; 15-items, Cronbach’s α = 0.59–0.9) [37] and the Implementation Climate Scale (ICS; six items, Cronbach’s α = 0.89) [38], as well as a brief demographics questionnaire. These measures were selected to provide descriptive information about the characteristics of individuals (i.e., providers’ attitudes) and inner setting (i.e., implementation climate) domains of the CFIR 2.0. The survey for patients contained brief demographic questions concerning their background and participation in the VeggieRx program, including two items that assessed food insecurity [39] and four items that assessed nutrition security [40]. We opted to assess food insecurity and nutrition insecurity in addition to participant demographics to better understand the food and nutrition characteristics of our sample and to assess whether the VeggieRx program was reaching the population it was intended to serve. Surveys took 5–10 min to complete and were administered immediately after the qualitative interviews. Results of the surveys will be presented in a separate manuscript.

#### Phase 1: procedures

##### Aim 1: formative evaluation

Prior to completing interviews, participants provided verbal consent to participate in study procedures. Trained research assistants who are fluent in English or Spanish were assigned to interview participants (providers/staff and patients) based on the participants’ language preference. Once study eligibility was confirmed, research assistants scheduled a time and date for the interview and brief post-interview quantitative measures. Participants were given the option of completing qualitative interviews via Zoom or in person, and all interviews were video- and audio-recorded via Zoom. Immediately following completion of interviews, participants were asked to complete quantitative measures via Qualtrics (self-report or interviewer administered, depending on participant literacy level and preference). Participants were compensated with a US $50 gift or debit card for their time.

#### Phase 1: Analytic plan

##### Quantitative

Descriptive statistics for the provider-level quantitative measures, including the EBPAS and the ICS, were calculated, including means, standard deviations, ranges, frequencies, and percentages. ICS scores were reported at individual and practice levels, assuming that practices had at least three providers or staff per practice completing the measure to maintain confidentiality.

##### Qualitative

Audio recordings of the interviews were transcribed and translated to English from Spanish to facilitate data analysis. Interviews were coded using a rapid analytic approach [41], in which a templated summary table was developed to summarize transcripts. The templated summary table included one column for each CFIR 2.0-informed interview guide question, including required probes, and one column for key points and exemplar quotes. Barriers and facilitators were coded using a priori deductive codes representing each of the CFIR 2.0 domains. These codes were described in a codebook that included a summary of each domain, a list of potentially relevant constructs, and specific examples from study interviews added in the early stages of coding. Within the templated summary table, there was one cell where coders could summarize barriers and facilitators for each CFIR 2.0 domain. Initial transcript summaries were generated by members of the analytic team; the study principal investigator conducted a secondary review of all summaries and discussed them with the analytic team to make any needed modifications and ensure consistency across coders. Final summaries were consolidated into matrices by participant type (i.e., patients and providers). The analytic team generated an exhaustive list of barriers and facilitators for the modified conjoint analysis method to be employed in Phase 2. In addition, the coding team reviewed matrices collaboratively and iteratively to identify themes and sub-themes across interviews, consistent with the framework method [42]. Weekly analytic team meetings provided an opportunity for discussion of coding and analysis to ensure consistency across team members. Discussion and consensus were used to resolve any questions and discrepancies in coding. Results were shared with the study team and participating sites.

### Phase 2 (Aims 2 and 3: developing and piloting an implementation blueprint for VeggieRx)

#### Phase 2: procedures

##### Aim 2: developing the implementation blueprint

Following the methods described by Lewis and colleagues [43], we developed an implementation blueprint using modified conjoint analysis. Conjoint analysis [44] is a method frequently employed in marketing to evaluate consumer preferences and priorities for specific product attributes. This method is particularly applicable to implementation blueprint development as it enables the prioritization and consideration of trade-offs for different implementation strategies [45]. Qualitative data from Aim 1 informed our understanding of VeggieRx implementation determinants, and we worked with our CAB to prioritize determinants and to assess matching implementation strategies. Specifically, in our first CAB meeting, we shared results from the qualitative interviews and asked CAB members to rate each barrier on feasibility to change (i.e., “high” or “low” feasibility) and importance to address (i.e., “high” or “low” importance). Prior to the second CAB meeting, the research team and representatives from Integra generated a list of implementation strategies drawn from the well-established ERIC project [30] and informed by existing literature [46] that could address the high feasibility and high importance barriers. This list of strategies was presented to the CAB at the second meeting for rating on feasibility (i.e., “high” or “low” feasibility of strategy use given existing resources). CAB members were then asked to rate each strategy in terms of its impact on VeggieRx progress success on a scale of “1 — low impact” to “3 — high impact.” Strategies were selected for inclusion in the blueprint if they were rated as high impact on fidelity (i.e., rating of 3), high feasibility, or both. After identifying strategies using this method, final strategies were selected and operationalized through discussion with CAB members in a third meeting. The list and operationalization of the final strategies selected for inclusion are provided in Table 3. These strategies were organized into a step-by-step blueprint document with strategies designated as supporting pre-implementation, implementation, and/or sustainment [47]. The fourth and final CAB meeting will involve receiving input from the CAB on pilot trial results (“member checking”) and planning for dissemination of findings.

**Table 3:** CAB-informed ERIC implementation strategies

**Barrier**	**ERIC**		**Action**	**Justification**
	***Category***	***Strategy***		
Logistical challenges	Provide interactive assistance	Provide local technical assistance	Integra develop and finalize referral processes and address logistical challenges	Integra ACO will run the VeggieRx program long term
Capacity	Adapt and tailor to context	Promote adaptability	Provide delivery of vegetables to all patients via 3rd party vendor (CartwheelRI)	Patients would find VeggieRx more acceptable and appropriate if VeggieRx offered delivery
Provider awareness	Train and educate stakeholders	Conduct educational meetings	Integra to conduct meetings with staff who facilitate program referrals to orient them to the referral process	Integra will facilitate the referral process, providing practices with updates
User-friendliness of vegetables/ Spanish language		Distribute educational materials	Distribute materials to patients (in English and Spanish) about VeggieRx and referral process	Patients receive a card or email with VeggieRx information
Provider awareness		Distribute educational materials	Distribute materials to providers about VeggieRx and how to make referrals	Distributing educational materials will help providers and staff learn about VeggieRx
User-friendliness of vegetables/Spanish language		Develop educational materials	Add tips about vegetable shelf-life, storage, and preparation to existing materials in both English and Spanish; include links to online videos/resources	Patients identified lack of familiarity with certain vegetables
Logistical challenges		Provide ongoing consultation (track strategies)	Schedule regular check-ins on the use of implementation strategies and troubleshoot/answer questions about challenges that arise related to employing the blueprint	Tracking implementation strategy usage is a critical component of implementation measurement
Provider awareness	Support clinicians	Remind clinicians	Champions to provide prompts to providers to screen patients for program	Providers may need prompts/reminders to ensure they remember to refer appropriate patients to the VeggieRx program
Logistical challenges	Engage consumers	Intervene with patients/consumers to enhance uptake and adherence	During intake process, assess patients’ needs related to social determinants of health (SDOH) and make referrals to resources that can address these needs	Ensure that patients receiving vegetable delivery appropriately and satisfactorily address their SDOH needs
Logistical challenges	Change infrastructure	Change record systems	Change to two-part referral system with integra relaying all patient information to southside	Methods to improve referral systems will likely help providers more easily make referrals to (adopt) the program

*Barrier*, identified by CFIR 2.0 qualitative interviews; *Action*, statements specifying discrete observable behaviors that encompass the implementation strategy; *Justification*, empirical, theoretical, or pragmatic justification for the choice of implementation strategies

*Abbreviations*: *ERIC* Expert Recommendations for Implementing Change, *SDOH *Social Determinants of Health

##### Aim 3: pilot study

Four primary care practices have committed to participate in this study and carry out implementation strategies identified in the implementation blueprint. Individual participants will complete informed consent procedures prior to participating. Patients (*N* = 10–15) and providers/staff (*N* = 10–15) will complete baseline surveys at the start of the 2023 vegetable delivery season, which begins in July, and again at 6-month follow-up. Patient baseline and follow-up measures (listed in Table 2) are each expected to take approximately 30 min and can either be completed via self-report or via interviewer. A biweekly measure of patient engagement will take approximately 2 min each time and will be sent via text message or email depending on participants’ preferences throughout the duration of the 6-month VeggieRx delivery period. All participants will be compensated by the study team with a US $50 gift or debit card for their completion of measures at baseline and US $75 at follow-up. Patient participants will receive compensation of US $3 for each week they answer the text or email survey, which will be paid at the end of the 6-month VeggieRx delivery period.

##### Implementation strategies

The “core components” [48] of the VeggieRx program, including primary care referrals to the program and patients receiving pre-packaged vegetables on a biweekly basis, will remain unchanged. However, implementation strategies [49] will focus on improving the extent to which VeggieRx is adopted, implemented, and sustained. Implementation strategies for this study will involve making changes to the adaptable components (“adaptable periphery”) [48] of the intervention (e.g., switching to delivery only) to improve its fit for the intended population and will also involve changes to the operations of the program (e.g., streamlining the referral process). With support from the study team, key players in the VeggieRx program (i.e., practices, Integra, SCLT) will begin to employ the implementation strategies defined by the implementation blueprint developed in Aim 2. For a 6-month period from baseline through follow-up, study staff will check in biweekly with staff at participating practices, SCLT, and Integra to collect details of implementation strategies being used. We will collect detailed information on individuals responsible for each strategy, specifics of strategies used, strategy targets (i.e., for whom the strategy was intended), timeline, and dose/frequency of strategy use. This approach to tracking implementation strategies aligns with the methods described by Rudd and colleagues and also includes identification of hypothesized mechanisms for each strategy [50]. Biweekly meetings with Integra will include consultation on the deployment of the strategies delineated in the implementation blueprint.

#### Phase 2: pilot participants

Healthcare providers and staff (*N* = 10–15) and patients (*N* = 10–15) will be recruited from the four practices to complete surveys asking about implementation and effectiveness outcomes. Inclusion criteria and sample size justification (i.e., likelihood of achieving saturation on qualitative interviews) for both types of participants are the same as in Phase 1. Patients and providers/staff who participated in Phase 1 will also be eligible to participate in Phase 2; additional participants will also be recruited to account for patient/staff turnover and to achieve planned recruitment numbers.

#### Phase 2: pilot recruitment

The same recruitment methods used in Phase 1 will be used in Phase 2 (see Phase 1 Recruitment section above). Patients who participated in Phase 1 will be eligible to participate in Phase 2, but we anticipate also recruiting new participants for this phase.

#### Phase 2: pilot measures

Study measurement will be guided by the CFIR 2.0 Outcomes Addendum [51], which delineates both implementation and innovation (effectiveness) outcomes. In addition, the Outcomes Addendum specifies several *antecedent assessments* or implementation determinants that have been shown to predict anticipated or actual implementation outcomes. These include acceptability, appropriateness, feasibility, implementation climate, and implementation readiness. In the present study, we intend to measure all five of these implementation determinants, as well as one preliminary implementation outcome (adoption), and three innovation (effectiveness) outcomes, as described below and shown in Table 2.

##### Antecedent assessments

*Acceptability *of the VeggieRx intervention will be measured with providers/staff at each site using the acceptability of intervention measure (AIM; four items, Cronbach’s α = 0.85) [52]. Additionally, acceptability will be measured with two open-ended written response questions that ask about providers’ perceptions of the feasibility and acceptability of selected implementation strategies. The use of open-ended response items was selected due to feasibility concerns related to scheduling qualitative interviews with providers and the importance of eliciting their feedback. Providers’ general *attitudes *toward evidence-based practices will be measured by the Evidence-Based Practice Attitudes Scale (EBPAS; 15 items, Cronbach’s α = 0.59–0.9) [37]. Biweekly surveys of engagement with the intervention will measure patient acceptability. Specifically, participants will receive a text message or phone call from study staff (based on their preferred method of communication) within a week of receiving each vegetable delivery. The survey will take approximately 2 min and ask the following questions: (1) Did you receive your vegetables last Friday? (yes/no); (2) Did you know how to use the vegetables? (yes/no with recipes included with the delivery); (3) How much did you like the vegetables included in your most recent delivery? (Likert scale: 1 *did not like at all* to 5 *liked very much*); (4) How many of the vegetables did you and your family eat? (Likert scale: 1 *used none* to 5 *used all the vegetables*); and (5) How much do the vegetables fit with the types of food your family eats? (Likert scale: 1 *not at all* to 5 *very much*). *Appropriateness *will be measured with providers/staff at each site using the intervention appropriateness measure (IAM; four items, Cronbach’s α = 0.91) [38]. *Feasibility *will be measured with the Feasibility of Intervention Measure (FIM; four items, Cronbach’s α = 0.89) [52]. We will also assess feasibility in terms of participant enrollment in the study and number of measures completed at each time point.

##### Implementation climate

We will assess the innovation-specific organizational climate for implementing produce prescription programs using the six-item Implementation Climate Scale developed by Weiner and colleagues (Cronbach’s α = 0.91) [38].

##### Implementation readiness

We will assess organizations’ (primary care practices') readiness for implementing change using the Organizational Readiness for Change Scale (ORIC; 12 items; Change Commitment Scale Cronbach’s α = 0.91; Change Efficacy Scale Cronbach’s α = 0.89) [53].

##### Implementation outcome

*Adoption* of VeggieRx will be measured from the web form used to enroll participants in the VeggieRx program. Specifically, we will assess patient adoption by measuring the number of patients enrolled in VeggieRx during the 6-month program period. We will assess provider adoption by noting the role/title of the person making each referral and the practice from which each referral was made. We will also assess practice-level adoption by noting the percentage of providers at each practice who referred patients at least once.

##### Innovation (effectiveness) outcomes

Behavioral/health outcomes will include the following: (1) food and nutrition security, (2) fruit and vegetable intake, and (3) depression symptoms. *Food security* will be measured by six-item USDA measure, and *nutrition security*will be measured by the four-item nutrition security measure [40]. *Fruit and vegetable intake *will be measured with the 10-item Dietary Screener Guide [54]. Finally, we will assess *depression *via the Patient Health Questionnaire-2 [55]. We will also administer a demographic survey to collect information on participants’ age, sex, race/ethnicity, number of members in the household, and zip code.

#### Phase 2: analytic plan

##### Quantitative

Descriptive statistics will be calculated (means, standard deviations, ranges, frequencies, percentages) for all outcome variables, including implementation (i.e., acceptability, adoption, appropriateness, feasibility, implementation climate, implementation readiness) and effectiveness (i.e., food security, body mass index, and depression). In addition to calculating descriptive statistics for standardized measures of implementation and effectiveness outcomes, we will also assess feasibility of study procedures based on participant enrollment. Specifically, we will assess the proportion of participants enrolled in the study relative to the total number of patients receiving VeggieRx, the proportion of participants enrolled in the study relative to the number approached to enroll in the study, and the proportion of participants who consent relative to the number who express interest in the study. Unadjusted effect sizes for differences from pre to post (e.g., differences in means, proportions) will be estimated for preliminary implementation (adoption) and effectiveness (food security, dietary quality, body mass index, depression) outcomes between baseline and follow-up. We will assess pre-post differences in adoption by comparing the number of vegetables shares provided, households served, and miss rates for pickups/deliveries in past years (2020, 2021, and 2022) and in the current year (2023).

##### Qualitative

Open-ended written response questions that assess the feasibility and acceptability of selected implementation strategies will be analyzed using a thematic framework analysis approach [42]. We will create one column for each question (i.e., one for feasibility and one for acceptability) and rows for each participant. Consistent with the example of data interpretation provided by Gale and colleagues [42, 56], we will use memoing to summarize the data in each column, and themes will be developed inductively based on provider and staff responses to each question.

### Power analysis

We did not conduct power analyses for this study given recommendations that power analyses are not appropriate for pilot studies that do not propose inferential tests [57]. Our sample size was determined based on the pragmatics of recruitment (i.e., including all sites currently involved with the VeggieRx program). Analyses instead focus on exploring descriptive data and examining the feasibility and acceptability of implementation strategies.

### Trial status

The Brown University Institutional Review Board has approved all study procedures. Phase 2 of the study is registered on ClinicalTrials.gov. Phase 2 participant recruitment began in June 2023 and is currently ongoing.

## Discussion

Despite the proliferation of produce prescription programs as a method for addressing food insecurity and diet-related illness [20, 58–61] and the evidence for their impact on vegetable intake and health-related outcomes [62], there is a need for additional research that explicitly examines methods to improve their implementation. Although some studies have begun to identify promising implementation strategies [46], few studies have explicitly developed tailored implementation strategies and examined implementation outcomes for a produce prescription program. Furthermore, few studies have meaningfully integrated community members’ perspectives in the implementation process [63]. In response to these needs, this study aims to assess existing determinants of VeggieRx implementation in primary care settings and use this information to inform the development of an implementation blueprint to improve implementation. Results will inform a future fully powered hybrid type 3 effectiveness-implementation study that will assess VeggieRx’s effectiveness for improving food insecurity and how successfully it can be scaled up in clinical practice settings.

This study has multiple strengths that directly address the limitations of the existing literature. Consistent with the 2022 Food is Medicine Action Plan’s [12] recommendation for research that seeks out perspectives and partnerships with community-based organizations, we will partner with multiple community organizations and convene a community advisory board to inform the development of an implementation blueprint. Implementation strategies will be developed using a systematic process responsive to identified barriers and tailored with input from our community advisory board. In addition, we will collect qualitative and quantitative data from multiple sources to understand implementation determinants (Phase 1) and to assess preliminary implementation and effectiveness outcomes (Phase 2). Data will be collected from patients, providers, staff, and our partners at Southside Community Land Trust to ensure that we consider a broad range of perspectives. Finally, our study is guided by a well-established implementation framework (the CFIR 2.0) to ensure that our implementation efforts consider determinants at multiple levels, ranging from individual to outer setting factors.

Despite these strengths, this study also has limitations. All sites will receive the same implementation strategies at the same time. Sites will not be randomized to receive strategies given the small number of sites involved at this time (*N* = 4). The goal of this study is to pilot the identified implementation strategies and assess preliminary effectiveness and implementation outcomes in preparation for a larger trial. Related to the small sample size and the scope of this pilot study, we will not have sufficient statistical power to conclude whether implementation strategies are effective; instead, we will examine means and effect sizes to assess whether our selected strategies are feasible and promising to address identified barriers. In addition, we are relying on self-report patient and provider data, which may be less accurate than observational or objective data. While there is a need to collect data from electronic health records on patient outcomes (e.g., BMI, blood pressure) and provider behavior (e.g., session notes), it was deemed to be unfeasible within the context of this study. We hope to develop infrastructure to use electronic health record data in future work. This study will focus on one specific produce prescription program in the state of Rhode Island; our hope is that findings from this study will generalize to other programs in the state and throughout the country; however, some of our findings may be specific to this program and our community partners.

In summary, this project will enhance the existing literature about implementation determinants of produce prescription programs by identifying barriers and facilitators to existing implementation and testing community-informed implementation strategies. Results from this study have the potential to have a larger health impact, especially on issues related to food insecurity and diet-related illness, by improving the reach and scalability of produce prescription programs. In addition, the Rhode Island Food Policy Council is convening all produce prescription programs in the state to better communicate and collaborate so that our future work can include statewide programs and compare enhanced implementation to implementation as usual.

### Supplementary Information

**Additional file 1.** Patient and staff interview guide.

## Data Availability

The qualitative dataset generated and analyzed during the current study is not publicly available due to the highly sensitive nature of interview transcript data. Publication of entire transcripts risk identifying research participants. Given the small dataset, quantitative data will not be made publicly available. However, de-identified quantitative data will be made available to individual investigators following a written request to the study principal investigators.

## Abbreviations

ACO: Accountable Care Organization
CAB: Community Advisory Board
SCLT: Southside Community Land Trust
VeggieRx: Vegetable prescription
