# Supplementary material for: Developing and testing a produce prescription implementation blueprint to improve food security in a clinical setting: a pilot study protocol
Source: Pilot Feasibility Stud. 2024 Mar 23;10:51. doi: 10.1186/s40814-024-01467-7 (PMC10960480; doi:10.1186/s40814-024-01467-7)
Supplement: Supplementary file 1 — Additional file 1. Patient and staff interview guide. [file 40814_2024_1467_MOESM1_ESM.docx]

**Appendix**

**Patient Interview Guide**

Introduction

*Hello! My name is* (insert name), *and I am a part of the* (insert affiliation with study*). Thank you for meeting with me today. Our research team is interested in your experiences with [VeggieRx from Southside Community Land Trust]* (use language the participant uses to describe the program)*. Your input is important because it will help us better understand what works and what doesn’t work with this program so we can make improvements.*

*I will be asking about your thoughts and opinions on how you get signed up for vegetables, the people and locations involved in this process, and if the program is accomplishing its goal. All responses are welcomed, and you are free to decline to answer for whatever reason at any time. Your opinion is valuable because it helps us understand how to make the program better in the future.*

*Our talk is expected to take 20 to 30 minutes to complete. With your permission, I will be recording our conversation to make sure I do not miss any important details and to focus on our conversation rather than me get distracted with writing it all down. Your responses will be kept private. Responses will not be connected to you in any product resulting from the research like papers or presentations. This recording will be destroyed three years after this study is finished. Do you have any questions before we begin?* (answer any questions)

*Okay, let’s get started. May I start the recording now?* (turn recorder on)

Background/Context

*Most of our conversation today will focus on the vegetable deliveries you get from Southside. Before we begin, what do you usually call this program?*

*Thank you. To start, tell me about your experiences with the program thus far.*

- OPTIONAL PROBES
  - How did you learn about the program?
  - Who connected you?

*Next, tell me what you think about the program.*

- OPTIONAL PROBES
  - How has the program compared to what you expected?
  - How much effort is required to participate in the program?
  - How has the program been helpful or unhelpful?
  - Do you like the food? How does it fit with what your family usually cooks and eats?
  - How have your food purchasing habits changed? For example, are you able to buy something else with the money you are saving from participating in the program?
  - How have your opinions about the program changed over time?

Facilitators and Barriers

*I’d like to move on to discussing the successes and challenges of participating in the program. Let’s begin with what worked well. What are some parts of it that you like, or that you think work well?*

- REQUIRED PROBES (If not discussed in response to initial probe)
  - What parts of the program are easy to access or use (e.g., delivery, signing up each season, etc.)? [**Implementation Process**]
  - Are there any people in your family or community who support your participation in the vegetable program? [**Individuals/Outer Setting**]
  - Is there anything about your doctor’s office or your medical providers that make it easy to participate in the vegetable program? **[Inner Setting**]
  - What positives have there been with the produce delivery, packaging, or quality? [**Innovation**]

*Now, what are some parts of the program that you don’t like, or that you don’t think work well?*

- REQUIRED PROBES (If not discussed in response to initial probe)
  - What parts of the program are hard to access or use (e.g., delivery, renewing prescription, etc.)? [**Implementation Process**]
  - Are there any people in your family or community that make it harder to participate in the vegetable program? [**Individuals/Outer Setting**]
  - Is there anything about your doctor’s office or your medical providers that make it hard or confusing to participate in the vegetable program? [**Inner Setting**]
  - What issues have there been with the produce’s delivery, packaging, or quality? [**Innovation**]

Final Questions

*As we wrap up, I’d like to get your thoughts on the program's future. How successful do you think this program will be moving forward?*

- PROBES
  - How motivated are you to participate in the vegetable program again?
  - Are you doing this program for your health? How does this program compare to other things you do for your health?

*What could we do to make the program better?*

- PROBES
  - What additional support should have been provided?
  - Are there programs that you think would work better?

Closing

*That brings us to the end of our interview. Do you have anything else you would like to share, or any questions for me before we wrap up?* (Answer any questions)

*Thank you very much for taking the time to share your thoughts with us. Your insight will help our team improve how the vegetable prescription program is delivered in the future.* (Stop recording).

*Now we ask that you complete a short survey. We can go through it together or you can take it on your own, whichever you prefer. We will send you a $50 gift card. We can offer you an Amazon gift card which we can send in an email or text or we can send you a visa card in the mail, as thanks for helping us with our study after we see that the survey has been completed. Which of these would you prefer?*

**Provider and Staff Interview Guide**

Introduction

*Hello! My name is* (insert name)*, and I am* (insert affiliation with study)*. Thank you for taking the time to speak with me today. Our research team is interested in your experiences with VeggieRx* (use whatever language participant uses)*. Your input will give us a better understanding about the challenges and successes with implementing this program so that we can make improvements.*

*I will be asking about your thoughts and opinions about VeggieRx, the people and locations involved in this process, and how well the program works. There are no right or wrong answers to these questions, and you are free to decline to answer for whatever reason at any time.*

*Our discussion is expected to take 20 to 30 minutes to complete. With your permission, I will be recording our conversation to help us make the best use of our time, but your responses will be kept private. Nothing you say during this interview will be linked to you in any related publications or presentations of this research. This recording will be destroyed three years after our study is finished. Do you have any questions before we begin?* (answer any questions)

*Okay, let’s get started. May I start recording now?* (turn recorder on)

Background/Context

*To start, tell me about your experience with VeggieRx.*

- OPTIONAL PROBES
  - What is your role in the program? (what facility/organization do you see your work in service of? Integra? Care NE? Your clinic?)
  - How/when were you introduced to the program?
  - Were you familiar with VeggieRx prior to when it was implemented at your facility/in this community?
  - What problems does the Veggie Rx program address?

*Did you have an implementation plan or follow any specific steps to start using VeggieRx in your practice?* [**Implementation Process**]

*Walk me through the process that a participant follows in your program.*

Facilitators and Barriers

*I’d like to move on to discussing the successes and challenges of implementing VeggieRx. Let’s begin with what works. Could you please describe some of the aspects of VeggieRx that you like, or that you think are going well?*

- REQUIRED PROBES (If not discussed in response to initial probe)
  - Are there components of the program that are easy to implement (screening, monitoring, etc.)? *Optional*: Are there any components of the program that you have added or improved upon since it first started? [**Innovation**]
  - What successes have you observed amongst program leaders or staff members? [**Individuals**]
  - Is there anything about your facility/work setting that can make it easy to implement VeggieRx? [**Inner Setting**]
  - Are there any patient-related factors that make it easier to provide VeggieRx? [**Individuals**]
  - Have local, state, or national policies or guidelines aided with implementing the program? [**Outer Setting**]

*Now, tell me about the aspects of VeggieRx that you don’t like, or that you don’t think work well.*

- REQUIRED PROBES (If not discussed in response to initial probe)
  - Are there components of the program itself that are hard to implement (screening, monitoring, etc.)? *Optional:* Are there any components of the program that you have not been able to start or had to remove since it first started? [**Innovation**]
  - What challenges have you observed amongst your program leaders or staff members? [**Individuals**]
  - Is there anything about your facility/work setting that can make it hard to implement VeggieRx? [**Inner Setting**]
  - Are there any patient-related factors that come up when trying to implement VeggieRx? [**Individuals**]
  - Have local, state, or national policies or guidelines interfered with implementing the program? [**Outer Setting**]

*How has COVID impacted the program?*

Final Questions

*As we wrap up, I’d like to get your thoughts on the future of the VeggieRx program. How prepared are you and your facility/organization to continue implementing the program?*

- OPTIONAL PROBES
  - What sort of feedback have you received from program participants about the program?
  - How do you anticipate costs or funding affecting the future of the program?
  - Does the program align with this facility’s/organization’s values and operations?
  - Do staff members receive clear direction or feedback on their role in program implementation?
  - How confident are you that you or other staff members can continue to make referrals/manage the program each season?
  - How motivated are you or other staff members to continue to make referrals/manage the program each season?
  - Do you and other staff members think this program is needed?

*Now that we have talked through what has made it easy or hard to implement the program, what improvements or additions would you like to see?*

- OPTIONAL PROBES
  - Are there any additional steps you feel should have been taken during the planning phase?
  - What tools would you like to have to help with implementing that you currently do not have?
  - Are there programs that you or other staff members think would work better?

Closing

*That brings us to the end of our interview. Do you have anything else you would like to share, or any questions for me before we wrap up?* (Answer any questions) *Are there any other providers/staff members who you think would like to share their thoughts with us?* (Wait for response)*. Great! Thank you very much for taking the time to share your thoughts with us. Your insight will help our research team modify VeggieRx to improve how it is implemented in the future.* (Stop recording) *Now we ask that you complete the post-interview survey. We can go through it together or you can take it on your own, whichever you prefer. We will send you a $50 gift card as thanks for helping us with our study after we see that the survey has been completed.*
